# Environmental DNA detects Spawning Habitat of an ephemeral migrant fish (Anadromous Rainbow Smelt: *Osmerus mordax*)

**DOI:** 10.1186/s12862-022-02073-y

**Published:** 2022-10-24

**Authors:** Vaughn Holmes, Jacob Aman, Geneva York, Michael T. Kinnison

**Affiliations:** 1 Center for Genetics in the Environment and School of Biology and Ecology, University of Maine, Orono, USA; 2 Wells National Estuarine Research Reserve, Wells, USA; 3 University of Maine Environmental DNA CORE Laboratory, Orono, USA

**Keywords:** eDNA, Rainbow smelt, Anadromous, Hierarchical occupancy models

## Abstract

**Background:**

Anadromous rainbow smelt (*Osmerus mordax*) have experienced a large range reduction in recent decades and the status of remnant spawning populations is poorly known in Maine, where these fish have significant ecological, cultural, and commercial relevance. Defining the remnant range of anadromous smelt is more difficult than for many declining fish species because adults are only ephemerally present while spawning in small coastal streams at night during spring runoff periods when traditional assessments can be unreliable or even hazardous. We hypothesized that eDNA might facilitate improved survey efforts to define smelt spawning habitat, but that detection could also face challenges from adult eDNA quickly flushing out of these small stream systems. We combined daytime eDNA sampling with nighttime fyke netting to ascertain a potential window of eDNA detection before conducting eDNA surveys in four streams of varying abundance. Hierarchical occupancy modeling was in turn employed to estimate eDNA encounter probabilities relative to numbers of sampling events (date), samples within events, and qPCR replicates within samples.

**Results:**

Results from the combined eDNA and fyke net study indicated eDNA was detectable over an extended period, culminating approximately 8–13 days following peak spawning, suggesting developing smelt larvae might be the primary source of eDNA. Subsequently, smelt eDNA was readily detected in eDNA surveys of four streams, particularly following remediation of PCR inhibitors. Hierarchical occupancy modeling confirmed our surveys had high empirical detection for most sites, and that future surveys employing at least three sampling events, three samples per event, and six qPCR replicates can afford greater than 90% combined detection capability in low abundance systems.

**Conclusions:**

These results demonstrate that relatively modest eDNA sampling effort has high capacity to detect this ephemerally present species of concern at low to moderate abundances. As such, smelt eDNA detection could improve range mapping by providing longer survey windows, safer sampling conditions, and lower field effort in low density systems, than afforded by existing visual and netting approaches.

**Supplementary Information:**

The online version contains supplementary material available at 10.1186/s12862-022-02073-y.

## Background

Documenting habitat occupancy is challenging for many organisms because of their behaviors, life histories, crypsis, habitat conditions, or rarity. One particularly challenging case is where organisms transiently occupy particular habitats for relatively short periods of time. For example, some migratory aquatic organisms may occupy breeding habitats for only a few days or weeks out of an entire year. Traditional survey methods that depend on direct encounters with such organisms may often prove ineffective, inefficient, or expensive due to high risk of missing these ephemeral events even with repeated site visits. Environmental DNA (eDNA), is quickly emerging as a sensitive and specific means of detecting many hard to survey species [1–4], but its utility for detecting some ephemerally-present organisms is unclear. Here we assess the utility and optimal survey effort for eDNA detection of a highly transient, stream breeding migratory fish, the anadromous rainbow smelt (*Osmerus mordax*).

Rainbow smelt are small fish that inhabit the northern temperate and arctic regions of North America and may exhibit either an anadromous or landlocked life history. This study focused on anadromous smelt that spawn in small coastal streams. Historically, smelt are important commercially and culturally as food, and ecologically as a forage fish for other species [5] The range of anadromous rainbow smelt formerly extended along the East Coast of the United States of America as far south as Chesapeake Bay, Virginia (~ 37.5214° N). However, the species southern range has retracted northwards to Buzzards Bay, Massachusetts (~ 41.7454° N). Some suggested causes of this decline include habitat degradation, dams, climate change and overfishing [6]. Even within their remaining coastal range, anadromous rainbow smelt appear to be in decline. However, quantifying that decline is difficult, as evidenced by the Maine Department of Marine Resources (MDMR) listing 46% of potential smelt spawning runs listed as “inactive” or “uncertain” in 2016 [7]. Low population abundances, complex life history and behavior of anadromous rainbow smelt, and difficult environmental conditions for visual or netting surveys are all factors in this challenge.

Smelt migration and spawning events typically take place over just a few nights in a given stream, during spring months (March-May) when rains and run-off make water conditions relatively high and turbid [6, 8]. As nocturnal spawners, adult smelt typically depart coastal streams by early morning, necessitating repeated nighttime visual surveys to encounter active spawning. Many visual surveys for smelt instead look for eggs left behind on rocks, which can be present for one week to one-month before larvae hatch and emigrate [5]. However, low abundance smelt populations leave behind relatively few eggs, and the eggs are not always easy to visually confirm [5]. Though traditional trapping surveys for adults can provide reliable population information, successful implementation of these surveys require significant investment of specialized gear and trained personnel time. We hypothesize that eDNA can improve smelt spawning habitat monitoring by providing increased detection sensitivity, a longer detection window, safer and more conducive survey conditions for crews, and less reliance on specialized field gear, by targeting the DNA shed by spawners or developing eggs and larvae.

eDNA approaches can improve on many traditional survey approaches by affording “sight unseen” detection [9] due to the aquatic environment’s propensity to distribute DNA shed from organismal tissues, fecal matter [10], and carcasses [11], for easy collection via water samples [9]. This capability may be especially beneficial when a target species is relatively rare in space or time, as is often the case for species of conservation concern or those establishing non-native populations [1, 3, 4, 12, 13]. However, eDNA has a limited period of availability once shed into a system due to processes like current transport, dilution, loss to sediments, and degradation [14–17]. Rivers and streams can present a particular challenge for eDNA detection because the flow in such systems is reported to quickly transport and dilute eDNA from a point source, with some estimates of detectable eDNA persisting only hours or days after the removal of a source [17]. This might seem to strongly limit the application window of eDNA for transient stream breeding organisms like smelt. However, while the breeding individuals may not be present in streams for very long, breeding activities like deposition of fertilized and unfertilized gametes, abrasion of tissues during nesting, or deposition of carcasses, may provide an increased window of opportunity [18].

To assess and refine the utility of eDNA for the monitoring of transient stream breeding rainbow smelt, we address the following questions:

1. Given the highly ephemeral nature of breeding smelt in streams, can they be detected using eDNA?
2. What is the window to detect smelt spawning beyond their active spawning window?
3. What sampling effort would be most effective for detecting low abundance smelt breeding in coastal streams, in terms of number of sampling events (dates) per stream, samples taken per date, and quantitative polymerase chain reaction (qPCR) replicates?

Addressing these concerns, we performed two field studies. The first compared smelt eDNA detection to fyke net catches, in two streams, which provide the answers to questions 1 and 2. The second field study built on the first using streams with known smelt spawning populations to answer question 3 via hierarchical occupancy modeling of empirical detections.

## Results

### Study 1: Fyke netting vs. eDNA

**Fig. 1:**
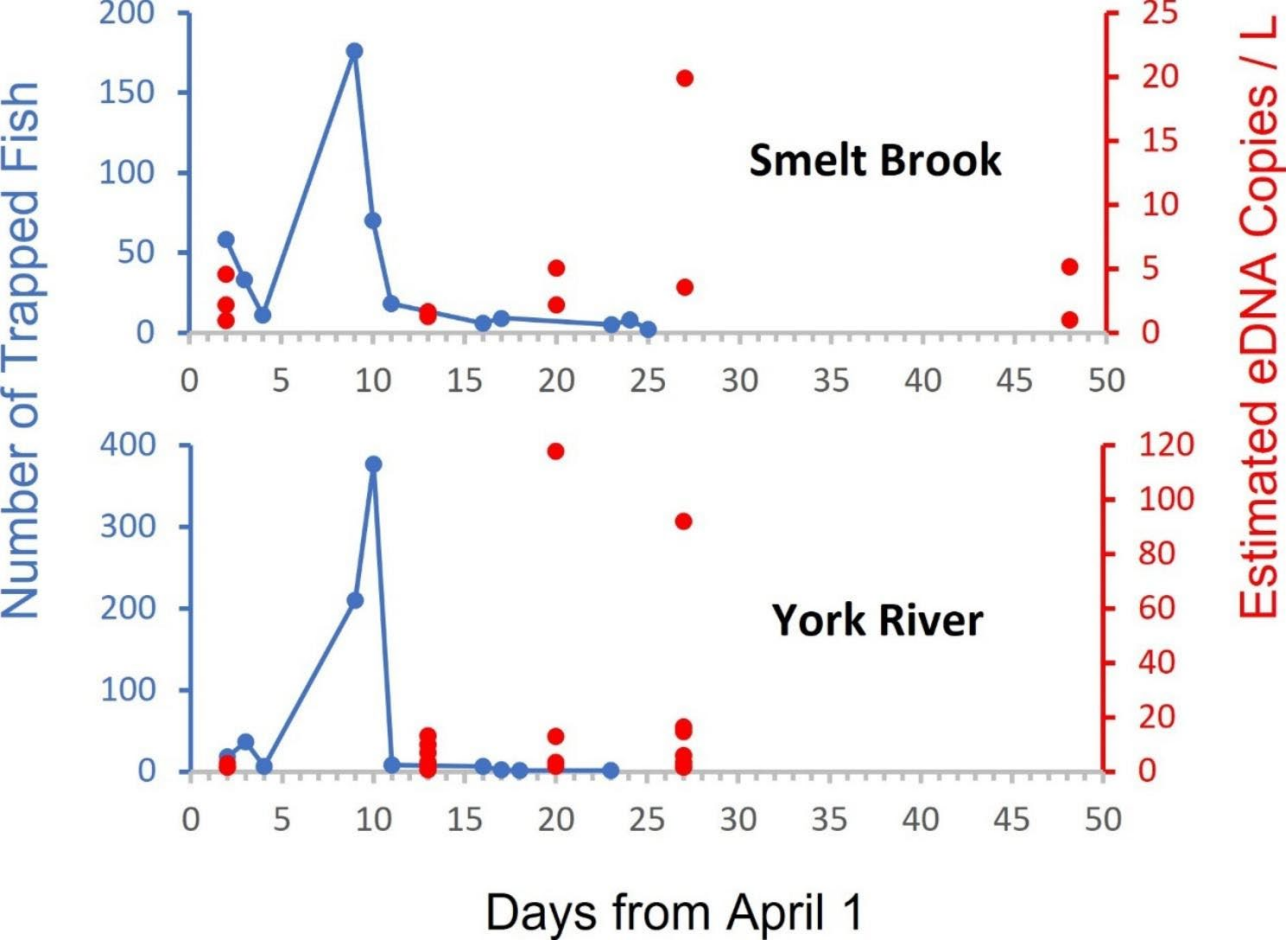
Study 1 Results: Adult smelt catch plotted with eDNA concentration (copies/L) for each PCR replicate at Smelt Brook and York River in April 2017 [20]

In April 2017 we paired eDNA sampling with an ongoing fyke net surveys at two sites in the York River system – the upper York River mainstem and Smelt Brook (a major tributary). Both fyke net sites experienced peak smelt catches around the 8-9th of April. However, while we detected eDNA throughout the suspected period of smelt spawning and incubation, we did not observe spikes in eDNA concentrations in samples collected closest to peak adult captures. Rather, highest eDNA concentrations (copies/L) occurred in samples 10–18 days after peak net captures (Fig. 1). This time interval corresponds well with regional smelt egg incubation periods [19] and informed sampling for the second study.

### Study 2: eDNA survey in four streams

**Fig. 2:**
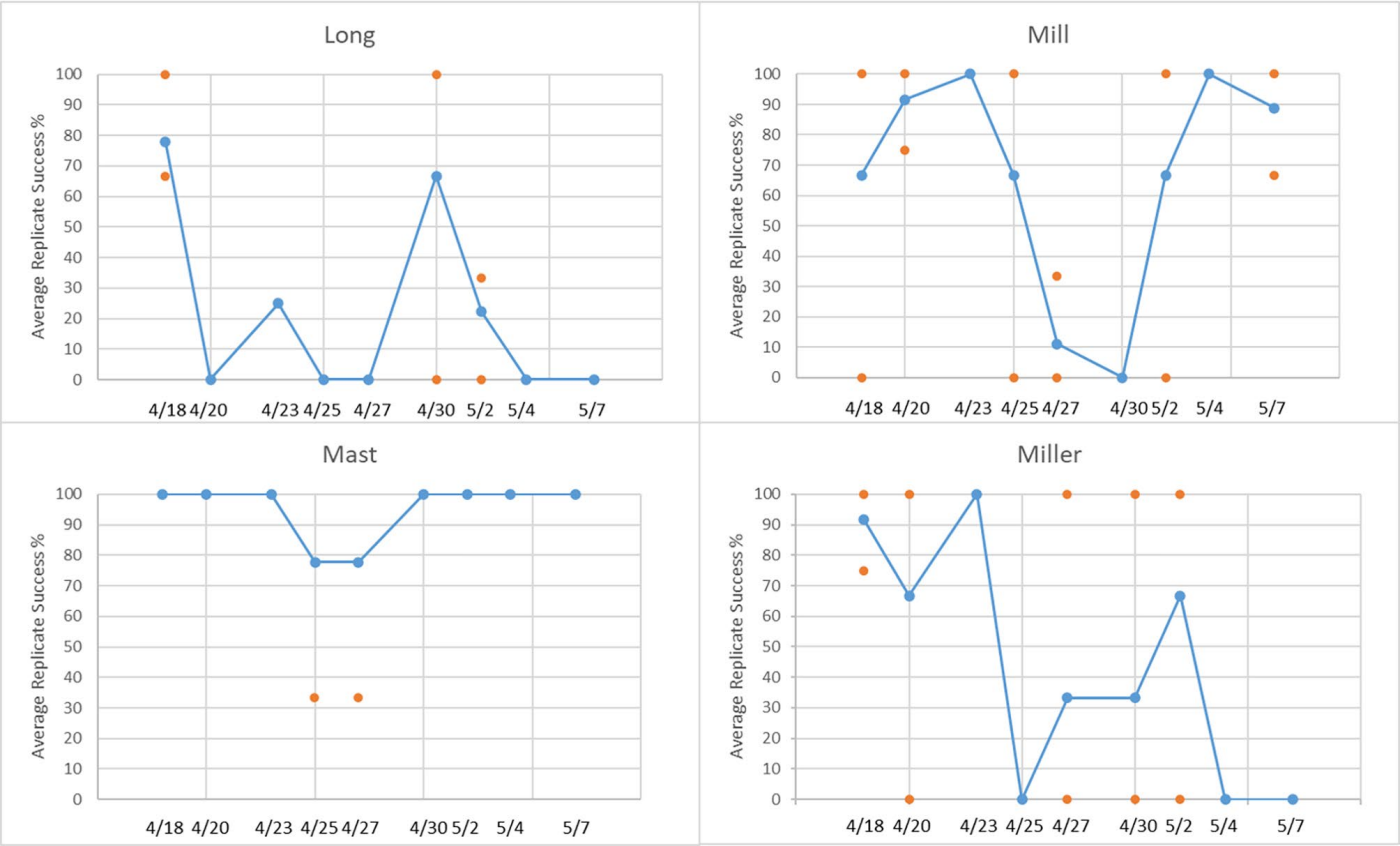
Study 2 Amplifications: Average percentage of successful amplifications (blue) out of 12 total replicates (dates 4/18 − 4/23) and 9 replicates (dates 4/25 − 5/7). Orange markers are the percentage of successful amplifications for individual samples on a given date, and depict sample-to-sample variability (note: some orange markers are concealed by other blue or orange markers). Due to laboratory complications, one sample (three replicates) is unaccounted for at Mast Landing on 4/25

In April-May of 2018 we conducted daytime eDNA surveys and visual egg surveys at four streams (Long Creek, Mill Creek, Mast Landing, and Miller Creek) identified by the Maine Department of Marine Resources as historically having high (n = 2, Mast Landing and Miller Creek) or low (n = 2, Long Creek and Mill Creek) smelt abundance [6, 21]. The high abundance streams were targeted as positive field controls whereas the low abundance streams were deemed more representative of systems to be targeted for future eDNA surveys. However, these classifications were based on historical data and ultimately one purported low abundance site (Mill Creek) had a greater percentage of successful eDNA amplifications than a high abundance stream (Miller Creek) (Fig. 2). qPCR efficiency was estimated at 99.70% (Additional File 1: Table S1, Fig S1). Negative controls were implemented both in the field and in the lab. Across all 108 negative control replicates only three amplified, two from Miller Creek on April 23rd (Cq = 38.96, 38.04) and one from Miller Creek on April 25th (Cq = 38.54). There were no instances of amplification in laboratory no-template controls.

### Occupancy modeling

Smelt eDNA was detected at all study sites, with the greatest number of positive dates, positive samples, and positive qPCR replicates, at Mast and Mill Creeks and lower numbers of positive detections at the Long and Miller Creek sites. Estimated occupancy probability per sampling event (days) at a given site ranged from posterior means of 0.66 (Miller) to 0.91 (Mast and Mill). Estimated per sample detection probability within dates ranged from posterior means of 0.67 (Long) to 0.89 (Mast). Estimated per qPCR replicate detection probability within samples ranged from posterior means of 0.41 (Long) to 0.96 (Mast). Mast Landing tended to have the narrowest (average width = 0.173) posterior credible intervals (PCI) averaged across all parameters; whereas Long Creek had the widest (average width 0.496). Average PCI width for all parameters (ψ = 0.44, θ = 0.27, p = 0.18) successively decreased from the highest tier parameter (occupancy) to the lowest (qPCR replicate) (Table 1).

**Table 1:** Occupancy Parameter Estimates: Posterior Means, Posterior Credible Intervals, and Copy Numbers

Parameter Estimates	Long	Mill	Mast	Miller
ψ (Day)	0.71	0.91	0.91	0.66
ψ (Day) PCI	0.39–0.97	0.69–0.99	0.69-1.0	0.36–0.91
θ (Sample)	0.67	0.73	0.89	0.73
θ (Sample) PCI	0.38–0.94	0.55-0.87	0.76-0.89	0.91–0.99
p(Replicate)	0.41	0.89	0.96	0.94
p(Replicate) PCI	0.25–0.60	0.82–0.96	0.91–0.99	0.86–0.99
AVG SCN/R	1.11	2.86	18.64	17.40
AVG SCN/L	9.30	23.88	155.38	145.03

The parameter estimates (means and 95% PCIs) for each level of the occupancy model are given along with estimated average starting copy number per reaction (SCN/R) and starting copy number per liter (SCN/L). ψ = sample event (days) occupancy probability, θ = per sample detection probability, p = Per qPCR detection probability

**Fig. 3:**
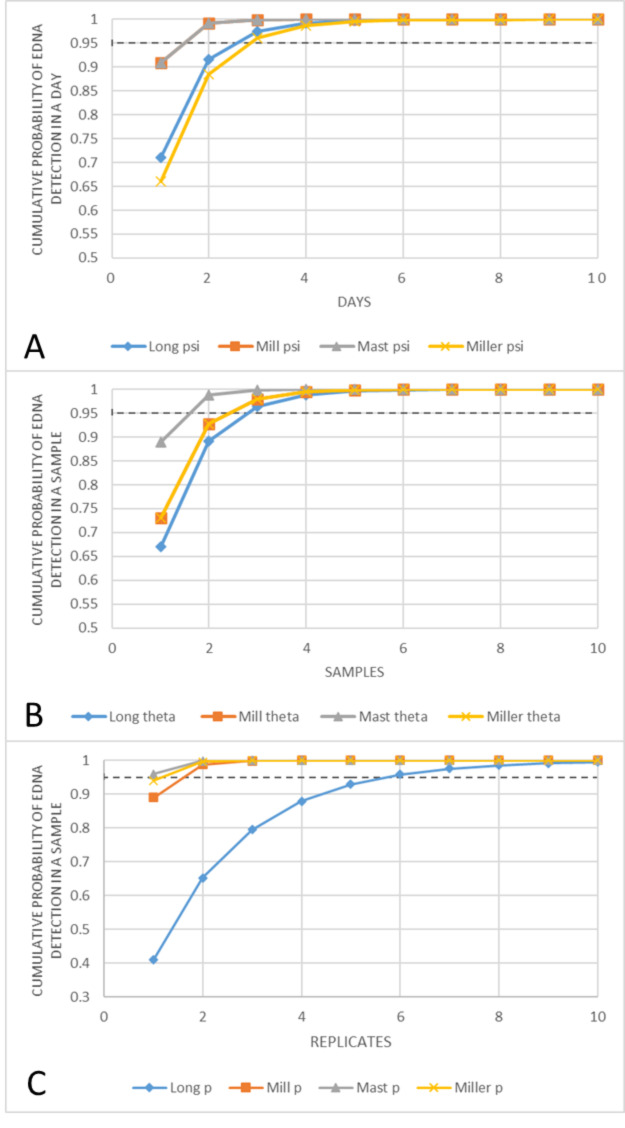
A-C: Smelt Cumulative Probability Functions: Cumulative probability functions derived from the occupancy model for each stream, separated by tier (A = Events, B = Samples, C = Replicates). The dashed line denotes a 95% probability of detection for the above values

As our study was focused on improving detection in low abundance streams, lower probabilities are more pertinent to establishing a robust sampling design. Using the site with the lowest value for each parameter, we in turn estimated the number of dates, samples and qPCR replicates required for a cumulative detection probability of 95% at each sampling level. These estimations are as follows: number of dates (ψ) = 3, number of samples (θ) = 3, and number of qPCR replicates (p) = 6 (Fig. 3 A-C). By comparison, our actual survey employed far more events per site (9), an equivalent number of samples (3), and about 33% fewer qPCR replicates per sample (4), achieving an estimated > 95% cumulative detection probability for all but the qPCR level at the Long Creek site

## Discussion

The goals of our paired studies were to: (1) Determine the viability of eDNA methods for detecting sea-run smelt, (2) Determine the duration of the detection window after active spawning, and (3) Determine what changes should occur to sampling effort to increase detection probabilities (p ≥ 0.95). Our findings demonstrate that anadromous rainbow smelt eDNA is detectable at low concentrations in coastal streams, even when samples are collected during daytime hours when adult fish have departed the system. Indeed, smelt eDNA was detected for weeks after peak spawning had passed. We believe this greatly increases the opportunity to efficiently and safely survey for these transient stream breeders when compared with traditional methods. We further demonstrate that the eDNA methods used had sufficient power to detect even very low abundance smelt populations, and that this power can be improved further with modest increases in sample processing effort (PCR replicates). These findings strongly support the role of eDNA sampling as a powerful tool for surveying anadromous rainbow smelt habitats, and we turn now to placing these findings into context of the biology of rainbow smelt, refinement of sampling design, and some added considerations for applying smelt eDNA assays more widely.

We found that rainbow smelt eDNA can be detected even after adult fish have departed streams. This is the case both on a daily spawning cycle and over the course of weeks following spawning. Anadromous rainbow smelt typically spawn at night in small streams and depart those streams by morning [5]. In our pilot study, eDNA samples were taken during the day, upstream of the fyke nets. As a result, it was unlikely that we detected eDNA from fish in the nets. Instead, it is likely that we detected holdover eDNA from several complementary sources that vary in importance over the spawning and post-spawning window. Notably, eDNA concentration did not strongly increase immediately following the peak spawning period of smelt, as inferred from fyke netting, and was never very high overall. This is consistent with most eDNA directly associated with adult fish being flushed from these short streams relatively quickly [17, 20]. However, some eDNA was retained in these systems, which might be attributed to three alternative sources—eDNA from deposited eggs, eDNA from carcasses, or eDNA bound in biofilms or sediments [11].

Carcass deposition can be an important source of eDNA in some anadromous fishes, such as semelparous Pacific salmon [18]. Anadromous rainbow smelt are not semelparous, but some mortality can be associated with spawning even in iteroparous species [6, 22]. Dead smelt were not directly observed in the streams during survey activities, but the small size of these fish makes it possible that a few carcasses could go undetected while decomposing over a period of days to weeks. Others have suggested that carcasses account for a smaller percentage of eDNA than living individuals in other species [23, 24]. Other studies [16] of eDNA production and loss have shown that eDNA can be bound in substrates and in turn remobilized under certain conditions [25, 26]. However, one would expect that detectable amounts of carcass and sediment-bound eDNA should decline over time following the peak of spawning activity, as these pools are gradually degraded or flushed from the system [24]. In contrast, we found evidence that peak eDNA concentration actually occurred 2.5–3.5 weeks following approximate onset of spawning (inferred from fyke net captures). This is similar to findings in a lentic system [27]. This leaves a final possible source of eDNA to consider – that deriving from developing fish eggs or larvae.

Although intact fish eggs are not apt to shed much eDNA, incidental death or predation on eggs could gradually release eDNA long after spawning. Indeed, deposited eggs should become richer sources of eDNA over time as embryos develop, with the greatest eDNA released close to and during hatching. For anadromous smelt, hatching occurs around 3 weeks after deposition in our study region [19], which closely coincides with peak eDNA concentrations observed in the first study. Larval smelt emigrate to sea quickly, so it also makes sense that eDNA values dropped off again in our study after about 4 weeks, when hatching was likely completed.

The findings from our initial fyke net study suggest that eDNA detection of smelt populations theoretically peaks around 2–3 weeks following spawning. In practice, however, it may be difficult to target sampling with such temporal precision in areas where anadromous smelt populations are poorly characterized. As such, it may often be necessary to distribute sampling effort across multiple dates to improve detection probabilities. Likewise, anadromous smelt eDNA was not detected in every sample or qPCR replicate in our second study. Because of the propensity for false negatives [28], we sought to determine how sampling effort might be best allocated across multiple sampling events, samples during such events, and qPCR replicates to provide high probability of detecting rainbow smelt spawning populations using both weak and strong spawning stocks. We in turn used these probabilities to generate cumulative probability functions for a given number of events, samples, and qPCR replicates (Fig. 3 A-C).

Assuming even the most conservative detection probabilities from study 2, we found a relatively modest level of sampling effort can achieve very high predicted detection. The results of our occupancy model suggested 6 qPCR replicates for a 96.06% probability of detecting smelt eDNA in a positive sample, 3 samples for a 96.40% probability of collecting eDNA when it is present on a given day, and 3 dates of sampling for a 95.78% probability of encountering smelt eDNA in a spawning system. This gives a combined conditional detection power of approximately 90% in any given year. These values overlap with other optimization studies applying alternative analyses [29] that account for false positive detections [30]. Our actual sampling effort matched or exceeded the projected efforts from our analyses for number of survey dates and samples per date, but we used 3–4 qPCR replicates as opposed to 6. Nonetheless, qPCR detection odds were projected to be high for all sites but Long Creek, where detection was still reasonable, at about 79% with 3 replicates.

Looking at stream-to-stream variation in cumulative detection probabilities suggests where effort is most needed in surveying for low abundance smelt populations and why. The number of sampling events or samples required to detect smelt when present did not vary much among sites, with three days or three samples per day providing > 95% probability of encountering smelt eDNA. This appears consistent with the biological processes giving rise to eDNA encounter rates in space and time. Both large and small smelt populations are expected to spawn in a very synchronized fashion in a given stream [31], even if that timing varies stream-to-stream, and we showed that eDNA detection persists for weeks after spawning, so it is reasonable that a relatively low number of sampling events spread over the season would be required for most systems. Likewise, the coastal streams studied here are all relatively small drainages (≤ 18.9km^2^) and smelt typically do not travel very far beyond tidal influence to spawn, both of which likely serve to reduce variability associated with sampling distances from spawning aggregations and the patchiness of eDNA often encountered in larger systems.

In contrast, there was a substantial difference between streams at the level of qPCR replicates. For most streams, the probability of detecting eDNA was over 90% per qPCR, indicating a need for as few as two qPCR replicates for > 99% probability of detecting eDNA in a positive sample. By comparison, the per qPCR detection probability for Long Creek was only 41%, suggesting 9 or more replicates would be required to achieve comparable power or 6 for > 95% detection. We suggest this substantial variation in power at the qPCR replicate level likely reflects the substantial influence that low population abundance and stream conditions can have on eDNA concentrations where it is encountered [32]. In other words, while smelt abundance might not have much influence on how smelt eDNA is distributed in space and time in these small streams, it could influence its concentration and likelihood of detection in a given qPCR reaction. Indeed, the eDNA concentrations in positive samples for Long Creek were lower than in all other streams (Table 2). Given the goal of improving documentation of declining sea-run smelt populations, it is important to highlight sampling needs for these lower abundance streams for future teams that may frequently encounter such populations.

Still, there may be cases where the aforementioned survey design is excessive or inadequate depending on a survey’s goals. For example, if the goal of the survey is to document whether a given stream is ever used by anadromous smelt, and streams will be surveyed in multiple years, then a lower survey effort might be acceptable in any given year with repeated annual opportunities to detect that population. Likewise, this suggested survey effort would not likely be adequate if the goal of a survey were to estimate precisely when smelt spawn in a system in a given year. It is probable that such a study would require sampling far more dates throughout the potential spawning season than would be required for merely documenting the presence of a spawning population. Given our first study results, doing so could further necessitate a back-calculation process to likely spawning dates based on peak eDNA concentrations, which brings many of its own complications.

Other guidelines can be followed to reduce some of the sampling intensity and analysis expenses of eDNA surveys for anadromous rainbow smelt. Although eDNA surveys can be more powerful than visual, angling, netting, or electrofishing surveys [4, 33, 34], that power isn’t always needed. Use of eDNA might be reduced or even entirely avoided in streams where smelt or smelt eggs are easily observed. At two of the sites in this study, Miller Creek and Mast Landing, eggs were observed by survey teams. We included these sites for the purpose of better understanding smelt eDNA detection, but in practice such sites could be immediately excluded from eDNA sampling or processing, saving processing costs. Likewise, if eDNA samples were processed quickly between site survey dates, or analyzed in batches starting with high probability dates, researchers could save considerably on processing costs by avoiding collection or analysis of redundant samples if and when eDNA is already sufficiently documented.

Our results showed some support for a relationship between average percentage of eDNA detections and expected abundances. Mast Landing (high abundance) and Long Creek (low abundance) represented the expected extremes, in terms of both percentage of detections and predictions made via occupancy models. The other two streams were nearly equal in most quantities and unexpectedly, Miller Creek (high abundance) had fewer detections overall and had more days with no detections, which resulted in the occupancy model suggesting an increase in days sampled. Notably, two of the three days with no detections were in the last two dates (May 4th 2018 and May 7th 2018) of the study. So, it is possible that smelt had left the stream and their DNA was flushed from the system prior to those dates.

Though ultimately smelt eDNA was successfully detected and verified in all four systems, we recommend following general field best practices, e.g., [35, 36] by including and documenting [37] positive and negative controls, including the types of positive controls (high abundance sites, synthetic gene fragments, and internal positive controls) employed in our design to ensure that baseline detection capabilities are comparable. Including known positive sites and synthetic smelt gene fragments provided strong confirmation that our field and lab approaches were functioning as planned. The inclusion of a commercial internal positive control nested in field water helped to identify where severe PCR inhibition was present. Almost no amplification of smelt DNA occurred in the second study before implementing a commercial inhibition clean-up step in our extractions. We did not test for inhibition in our first study, but eDNA detection rates and concentrations were substantially lower in the first study compared to the second. Inhibition, as well as smaller sample volumes (doubled from 1 to 2 L in the second study), could explain these differences [38]. Indeed, variable inhibition could provide an alternate explanation for changes in eDNA concentrations over the survey season in the first study.

To mitigate false detections, our smelt assay was designed so as not to amplify off-target species likely to be present in the same or upstream habitats. Negative control samples were applied at field, filter and qPCR levels, to monitor for contamination. In the second study DNA was amplified in three negative field controls, but not in ways that would affect our study findings. Two of these controls were from the same site/date were associated with implied eDNA concentrations (avg Cq = 38.5) that were far lower than in actual positive samples (Avg Cq = 29.1). The other control amplified with a Cq value of 38.54 was from a site/sample set that did not have any actual sample amplifications.

Though not a factor for our particular study sites, surveys in coastal streams with headwater lakes supporting landlocked rainbow smelt populations could encounter detections from the non-target landlock populations. If there is some uncertainty of that potential for a given stream, then survey teams should take samples of eDNA upstream of putative anadromous smelt spawning habitats to confirm absence of landlocked smelt eDNA. We also caution that the survey design we have recommended here was designed for small coastal streams. Anadromous smelt can spawn in much larger rivers and a subsequent study would be needed to determine the appropriate survey design.

## Conclusion

At present, approximately 131 anadromous smelt spawning locations in Maine have uncertain or inactive status and the species has been in decline throughout the region [6]. Regularly and reliably surveying these habitats is a daunting prospect with traditional tools given the transient spawning biology of the species and challenging observation conditions. With our 3 questions answered, it is evident that eDNA can be used to detect rainbow smelt at low abundances for days to weeks following spawning events, greatly expanding the capacity for high-power surveys of smelt status [39]. Moreover, smelt eDNA sampling can be conducted during the daytime and without the need to net fish, or disturb eggs, making this approach more cost effective, and safer for both survey teams and low abundance smelt populations.

## Methods

### Development of primer and probe set

We targeted the mitochondrial ND5 gene for primer and probe design because of the high copy number of mitochondrial genes and taxonomic specificity of this locus [12]. Sequence data for rainbow smelt was obtained from GenBank (www.ncbi.nlm.nih.gov) and aligned using the Benchling software [40] with homologous sequences for 12 other freshwater fishes that overlap in stream or lake habitats in Maine. There are no other osmeriform fishes in Maine streams. Based on these alignments, we identified a 134 bp fragment for development of a TaqMan MGB-NFQ qPCR assay using 6-FAM as a fluorophore and a 3’ non-fluorescent quencher. This assay resulted in a minimum of 8 bp (36.36%) mismatches for the forward and reverse primer and 5 (22.72%) in the probe when compared to the off-target species (Table 3, Additional File 2: Table S2). We confirmed in silico specificity of this marker set using Primer BLAST against all available sequences in the NCBI database.

**Table 3:** Stream Summary: All streams observed in studies 1 and 2

Study/Stream	ID	Town	Latitude	Longitude	Context
1/Smelt Brook	SMB	York	43.1796490	-70.7349330	Fyke Netted/eDNA-Strong Run
1/York River	York	York	43.1572610	-70.7372680	Fyke Netted/eDNA-Strong Run
2/Long Creek	Long	South Portland	43.633270	-70.333263	Egg surveys/eDNA-Weak Run
2/Mill Creek	Mill	Falmouth	43.731386	-70.225159	Egg surveys/eDNA-Weak Run
2/Mast Landing	Mast	Freeport	43.859627	-70.0833356	Egg surveys/eDNA-Strong Run
2/Miller Creek	Miller	Brunswick	43.8611889	-69.975642	Egg surveys/eDNA-Strong Run

Context indicates how each stream was sampled and the relative strength of the rainbow smelt spawning run.

Following in silico design and testing, lab testing was conducted using DNA extracted from fin clips of smelt and the other common Maine fish species (Table 2). Tissue samples were extracted via DNeasy blood and tissue kits (Qiagen) using the manufacturer’s protocol. Amplification was initially tested with standard PCR under the following conditions: 95℃ for 7 min, (95℃ 30 s, 60℃ 30 s, 72℃ for 90 s) x 30 cycles, 72℃ for 7 min. The final marker set, was tested on smelt and other fish DNA for sensitivity and specificity using the following conditions: 95℃ for 10 min, (95℃ 15 s, 60℃ 15 s) x 50 cycles

## Sites and sampling

As noted above, the goal of our first study was to assess whether smelt eDNA could be detected and over what post-spawning time window. For this purpose, we paired smelt eDNA sampling with fyke net surveys in the York River and Smelt Brook. The fyke netting portion of the survey, conducted by the Wells National Estuarine Research Reserve (WNERR), is detailed in the report “An Assessment of Spring Fish Communities” [20]. Briefly, fyke nets with wings and a first chamber of 0.64 cm mesh, and subsequent chambers of 0.32 cm mesh were deployed between early April and the first week of June. Nets were set in the thalweg of each stream, with the opening facing downstream and net wings extended across two-thirds of the channel. Nets were left to fish overnight for three successive 24-hours periods each week for the duration of the study. The catch was checked at low tide in each 24-hour period when water levels were at their lowest to aid access to the nets.

**Fig. 4:**
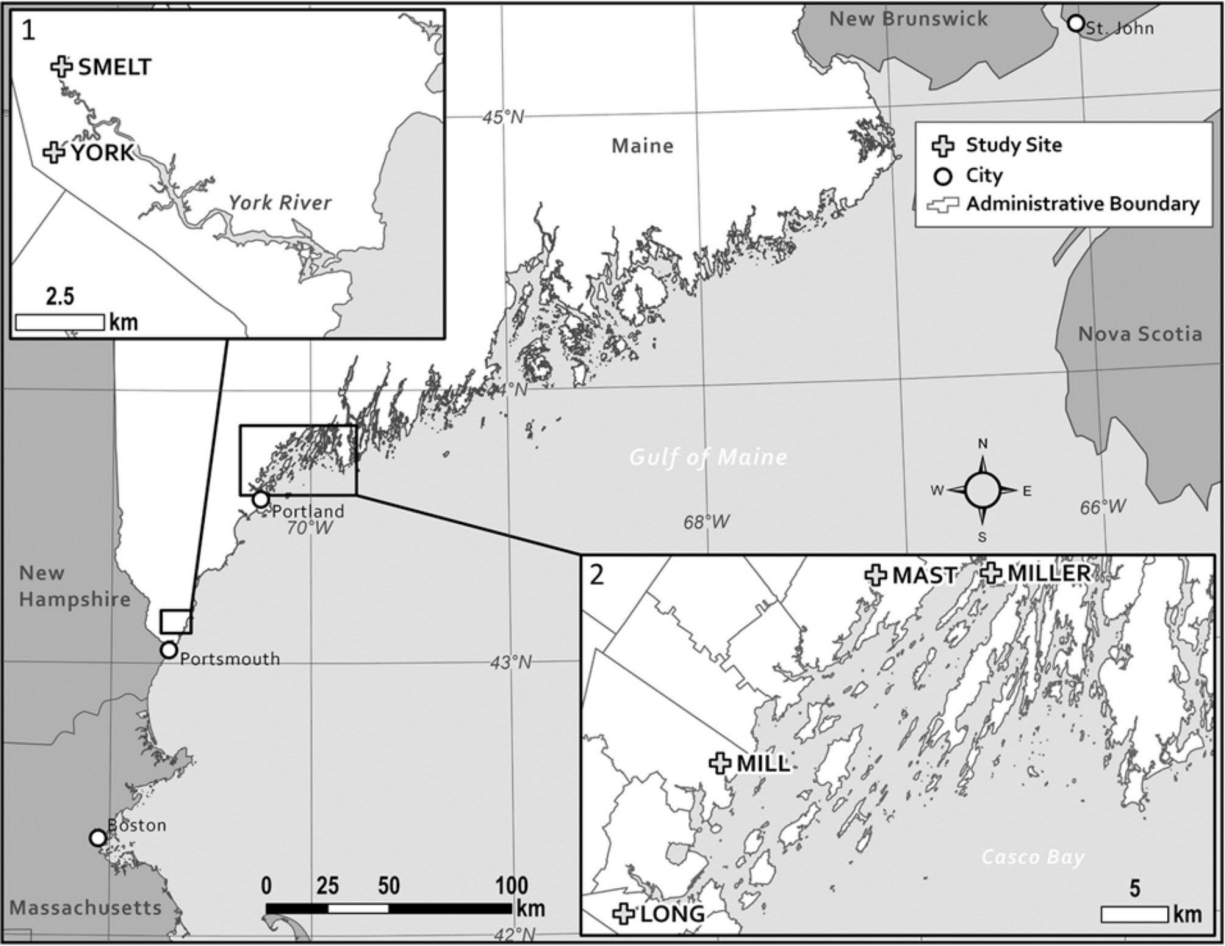
Map of surveyed streams: Sites for both studies were located in Southern Maine. Study 1 was centered around the York River, while study 2 streams were centered around Casco Bay

**Table 2:**
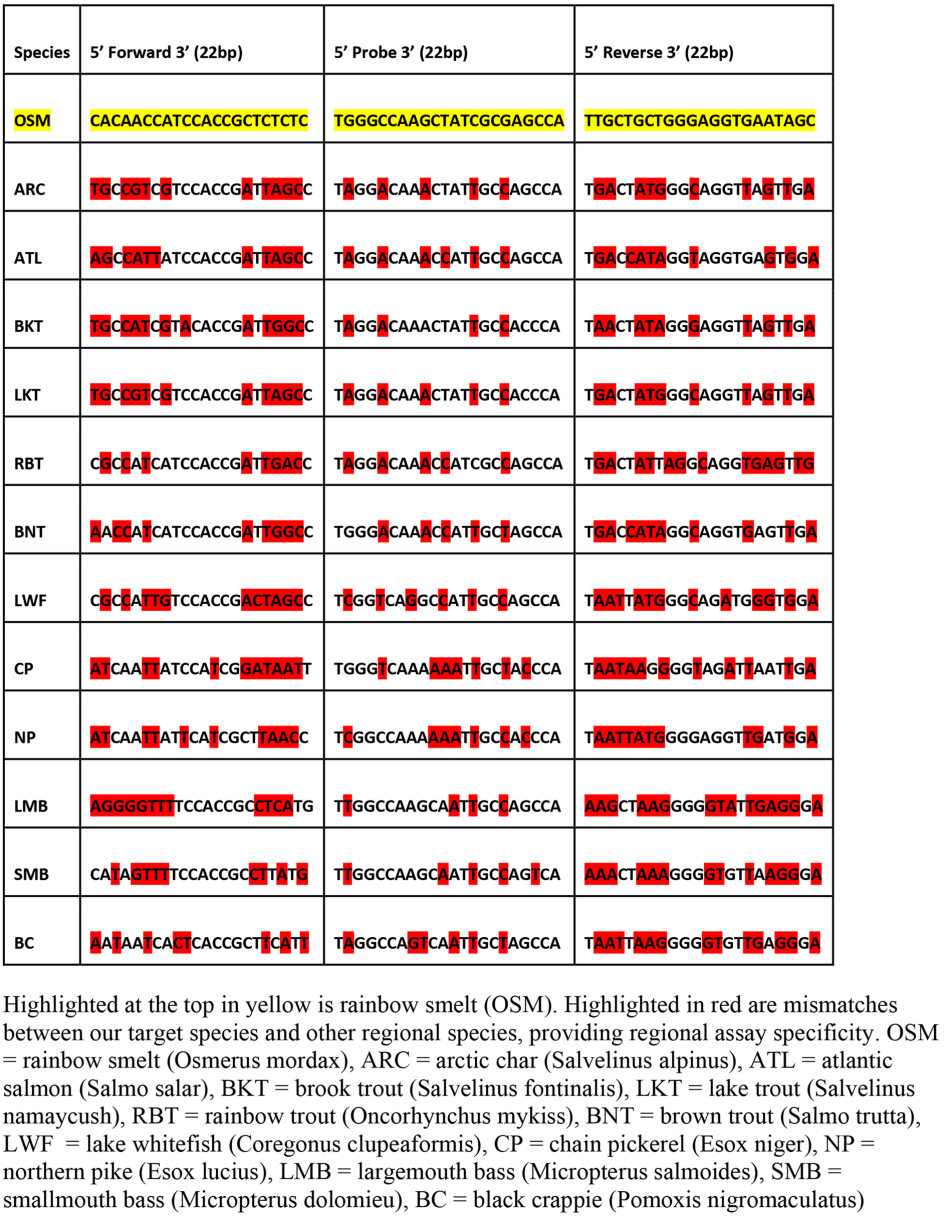
Smelt NAD5 TaqMan MGB-NFQ qPCR Primer-Probe Set: Sequence Alignment and Off-target Mismatches

Paired eDNA samples for this study were collected during the day on five non-consecutive dates spanning from April-May of 2017 (Apr 3rd, Apr 14th, Apr 21st, Apr 28th, May 19th ) at the York River and Smelt Brook Sites (Fig. 4). For each sampling event, eDNA sampling kits were prepared in a clean lab space to keep supplies free of contamination. Each sample kit consisted of a bag (Ziplock) large enough to contain two 500 mL water bottles (Nestle Pure Life) on the first two days and one 500mL water bottle on the remaining days. Four of these kits were prepared per site, three acted as field samples and one (500mL kit) as a negative control We also prepared a separate bag to hold gloves, assembled all of these materials in a larger, clean, trash bag for each site, to keep the sampling gear free from contamination during transport. A bleach-treated cooler was used in taking kits to and from each site. On each day, three samples were taken just upstream of and prior to setting the fyke net at low tide. One sample was taken at the right, left, and center of a given stream. A negative field sample control was collected at each site by opening a water bottle then closing it. Once collected, the samples and the control were placed back into their labeled Zip-lock bags, separated by site in closed trash bags, and transported back to WNERR in an ice cooler. Samples were then frozen at -20 °C, which is beneficial for eDNA recovery if samples are not processed within two weeks [41]. Samples were subsequently thawed and filtered at the University of Maine.

The second component of the study was to evaluate and refine the protocol for smelt detection, using eDNA, in sites of varying population density. Based on Department of Marine Resources (DMR) observations from 2005 to 2009 and 2012–2014, we selected sites in Long Creek, Mill Creek, Mast Landing, and Miller Creek (Fig. 4; Table 2). In 2018 environmental DNA was sampled near low tide on 15 dates between March 29th and May 9th, equating to roughly every 2–3 days. Nine of these dates (Apr 16th - May 6th ) were subsequently analyzed for this part of the study based on visual confirmation of the period when eggs were present at regional spawning areas. We also increased the volume per sample to 2 L (4 × 500 mL bottles) for each of the right, center, and left channel samples along with the negative field control. Due to its small size, the three samples collected on a given date at Miller Creek were sampled from downstream to upstream at intervals of approximately 2 m. All samples were collected near low tide in areas of moving water, avoiding pools and eddies, to minimize tidal mixing. Again, samples were frozen at -20 °C until filtration at the University of Maine or WNERR.

## In-lab procedure

Samples and field controls were vacuum filtered through 1.5 micron pore size 47 mm diameter glass microfiber filters (Whatman). The eDNA filter apparatus was sanitized with 10% bleach solution and rinsed with DI water between samples. Filtering spaces were sanitized before and after use with a combination of 10% bleach solution and UV light (60 min). The filters were then frozen at -20℃ for no more than two weeks before DNA extraction. If it was known extraction could not be accomplished in two weeks, samples were stored at -80℃ to further delay degradation of DNA. Extraction was undertaken using Qiagen DNeasy Blood and Tissue kits following a modified protocol (Additional File 3). In this extraction process, filters were not pooled and therefore each of the three samples taken at a site was treated independently. A ZYMO Research OneStepTM-PCR Inhibitor Removal kit (D603) step was included in order to reduce the burden of PCR inhibitors observed in pilot amplifications

qPCRs of samples were conducted on a Bio-Rad CFX96 Real-Time System in a 96-well PCR plate format. Each extracted sample and cooler blank was run with 3–4 technical replicates with assay concentration of 1µM primer and 500nM probe using the following chemistry: 10 µl TaqMan Environmental Master Mix 2.0 (Applied Biosystems), 5 µl nuclease free water, 2 µl of primer/probe/nuclease free H_2_0 mix, and 3 µl of extracted template, for a total reaction volume of 20 µl. A no-template control was similarly replicated on each plate, but substituted DNA-free water for the template. Positive controls in the form of a dilution series of six known concentrations of synthetic target DNA (Gblocks) were included to provide a standard curve for estimating starting copy numbers of eDNA and testing assay efficiency. An internal positive control (TaqMan™ Exogenous Internal Positive Control Reagents) was run in environmental samples and positive/ negative control wells for all but three dates (Apr 18th - Apr 23rd ), to facilitate detection of PCR inhibition. qPCRs were run under the following conditions: 95℃ for 10 min, (95℃ 10 s, 60℃ 30 s)x 47 cycles

## Analysis

Efficiency curves were estimated by analysis of covariance of log of the synthetic gene fragments of known concentration ((10, 50, 250, 1250, 6250, 31,250) copies/µl) against their corresponding quantitation curve (Cq) value. Efficiency was calculated as,

E = -1 + 10^(−1/slope)^.

Where the slope of the standard curve generated should be equal to -3.32 for 100% efficiency [42].

Environmental DNA concentrations per reaction were estimated from the qPCR fluorescence curves using the synthetic gene standard calibration curves. Subsequently, these reaction concentrations were volumetrically converted to copy number per liter based on extraction volumes. In the first study, copy numbers of individual PCR replicates were plotted with total captured adult smelt. Plotting these values over the days they were sampled allowed for a comparison of peak catch and peak eDNA concentration. In the second study, one copy number estimate per site was obtained by averaging all replicate copy number values. Zeroes(non-amplifications) were excluded from these averages.

With data from the second study (Additional File 1: Table S3(A-D)), hierarchical occupancy modelling was conducted in R (Version 4.0.3) using eDNAoccupancy package [43] for fitting multi-scale, occupancy models in a Bayesian framework without false positives. This package utilizes a space-state model composed of two main equations. The first is a binary occupancy state which represents smelt DNA presence or absence during a given sampling event (date) (i). The second equation is dependent on the original binary occupancy state which represents smelt DNA presence or absence in a sample from a given event (j). An analogous equation was applied once again for a third tier of the model (k). This tier corresponds to smelt DNA presence or absence within a qPCR replicate of a sample [44].

1. Z_i_ ~ Bernoulli (ψ_i_) for i = 1,2,…N.
2. µ_ij_ǀ Z_i_ ~ Bernoulli (θ_ij_) for j = 1,2,… V.
3. y_ijk_ǀ µ_ij_ ~ Bernoulli (p_ijk)_ for k = 1,2,…S.

Where: ψ = Number of sampling events(days), θ = Number of samples taken per day, p = Number of replicates per sample for a given day. For each tier, cumulative probability was calculated for a power analysis of the sample design. Cumulative probability was calculated as.

x*=1-(1-x)^n^ where x = ψ, θ, or p and n = i, j, or k.

depending on the tier of the hierarchy. In our studies 11,000 iterations of the Markov Chain Monte Carlo (MCMC) algorithm was used to fit the occupancy model and each site was fitted separately. Thus, our resulting outputs per site were, ψ = sampling event detection probability ,θ = sample collection detection probability, and p = qPCR detection probability.

## Electronic supplementary material

Below is the link to the electronic supplementary material.

Supplementary Material 1

Supplementary Material 2

Supplementary Material 3

## Data Availability

Data supporting the efficiency of the assay and occupancy model can be found in Additional File 1.

## Abbreviations

eDNA: Environmental DNA.
SCN: Starting Copy Number.
WNERR: Wells National Estuarine Research Reserve.
PCI: Posterior Credible Interval.
qPCR: Quantitative Polymerase Chain Reactions.
Cq: Quantification Cycle.

## Declarations

All experimental protocols were approved by the York River Wild and Scenic Study Committee and collection and handling of fish was granted by Wildlife Study Committee and Maine Department of Marine Resources (SPECIAL LICENSE NUMBER ME 2017-26-01).

No live vertebrates were handled for the eDNA project reported on in this paper.

All methods were carried out in accordance with Wells National Estuarine Research Reserve guidelines.

We state that the methods were reported in accordance with ARRIVE guidelines.

We do not have consent to participate issues because this was not human subjects research.
