# Correction: Environmental DNA detects Spawning Habitat of an ephemeral migrant fish (Anadromous Rainbow Smelt: *Osmerus mordax*)

**DOI:** 10.1186/s12862-023-02114-0

**Published:** 2023-06-01

**Authors:** Vaughn Holmes, Jacob Aman, Geneva York, Michael T. Kinnison

**Affiliations:** 1 Center for Genetics in the Environment and School of Biology and Ecology, University of Maine, Orono, USA; 2 Wells National Estuarine Research Reserve, Wells, USA; 3 University of Maine Environmental DNA CORE Laboratory, Orono, USA

**Correction** **:** **BMC Ecology and Evolution (2022) 22:121** 10.1186/s12862-022-02073-y

Following publication of the original article [1], the authors would like to correct the dates in the third paragraph under the heading **Sites and sampling**.

The sentence currently reads:

Nine of these dates (Apr 16th–May 6th) were subsequently analyzed for this part of the study based on visual confirmation of the period when eggs were present at regional spawning areas.

The sentence should read:

Nine of these dates **(Apr 18th–May 7th)** were subsequently analyzed for this part of the study based on visual confirmation of the period when eggs were present at regional spawning areas.

Moreover, the authors identified an error in Fig. 2. The correct figure (Fig. 2) is given in this correction.

**Fig. 2:**
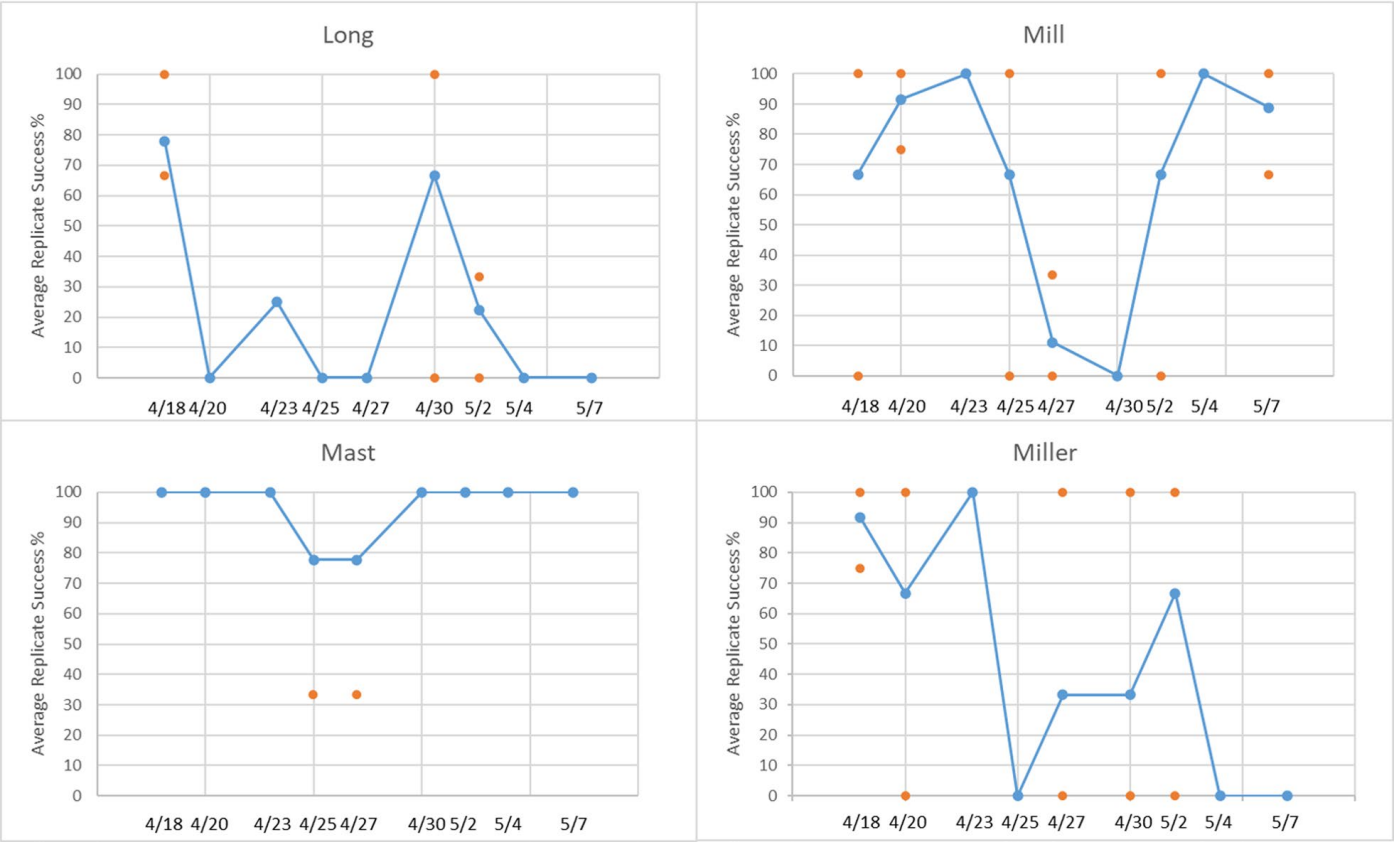
Study 2 amplifications: average percentage of successful amplifications (blue) out of 12 total replicates (dates 4/18−4/23) and 9 replicates (dates 4/25−5/7). Orange markers are the percentage of successful amplifications for individual samples on a given date, and depict sample-to-sample variability (note: some orange markers are concealed by other blue or orange markers). Due to laboratory complications, one sample (three replicates) is unaccounted for at Mast Landing on 4/25

The original article [1] has been updated.

## References

[1] Holmes V, Aman J, York G, Kinnison MT (2022). Environmental DNA detects Spawning Habitat of an ephemeral migrant fish (Anadromous Rainbow Smelt: *Osmerus mordax*). BMC Ecol Evol.

