# Supplementary material for: Environmental DNA detects Spawning Habitat of an ephemeral migrant fish (Anadromous Rainbow Smelt: Osmerus mordax)
Source: BMC Ecol Evol. 2022 Oct 24;22:121. doi: 10.1186/s12862-022-02073-y (PMC9594880; doi:10.1186/s12862-022-02073-y)
Supplement: Supplementary file 1 — Supplementary Material 1 [file 12862_2022_2073_MOESM1_ESM.docx]

**Additional File 2**

**Table S2**. List of Genbank accession numbers corresponding to table

| Species | Accession |
| --- | --- |
| **OSM** | AF034752.1 |
| **ARC** | AF154851.1 |
| **ATL** | JQ390055.1 |
| **BKT** | AF154850.1 |
| **LKT** | Locally Sequenced |
| **RBT** | NC_001717 |
| **BNT** | NC_024032 |
| **LWF** | JQ661483 |
| **CP** | AP013046 |
| **NP** | AP004103.1 |
| **LMB** | DQ536425.1 |
| **SMB** | NC_011361.1 |
| **BC** | NC_028298.1 |
