# Supplementary material for: Environmental DNA detects Spawning Habitat of an ephemeral migrant fish (Anadromous Rainbow Smelt: Osmerus mordax)
Source: BMC Ecol Evol. 2022 Oct 24;22:121. doi: 10.1186/s12862-022-02073-y (PMC9594880; doi:10.1186/s12862-022-02073-y)
Supplement: Supplementary file 2 — Supplementary Material 2 [file 12862_2022_2073_MOESM2_ESM.docx]

**Additional File 3**

**A. PURPOSE**

The purpose of this standard operating procedure (SOP) is to describe the procedures required to use DNeasy® Blood and Tissue Kit for the extraction of eDNA from filters.

**B. SAFETY PRECAUTIONS**

- Use standard laboratory PPE
- Use sterilized, filtered pipette tips
- Ensure centrifuge is properly balanced

**C. EQUIPMENT AND MATERIAL REQUIRED**

1. Laboratory PPE
2. DNeasy® Blood and Tissue Kit
   - Proteinase K
   - Buffer ATL
   - Buffer AL
   - Buffer AW1
   - Buffer AW2
   - Buffer AE
   - 2 ul collection tubes
3. Qiagen Investigator Lyse & Spin Basket kit
4. 1.5 ul microcentrifuge tubes
5. Sterile forceps (1 per filter)
6. Hazardous waste bags/collection vessel
7. Fine tipped marker
8. Timer
9. Pipette(s)
10. Sterile box of filtered pipette tips for each size of pipettes used
11. Shaking incubator
12. Microcentrifuge
13. Vortexer
14. bleach wipes
15. 50% bleach solution
16. Kim Wipes
17. DNA-off
18. UV sterilization light
19. 100% ethanol
20. Gloves

**D. PROCEDURES**

1. **Be advised: This SOP will not tell you to change your gloves constantly, but you should. If your glove touches any liquid that contains DNA, change them.**
2. **Be advised: Incubation times are suggested, but may be changed. They MUST be standard across all samples to be compared. This includes digestion and elution incubations.**
3. **Clean the work station with Clorox Bleach wipes and DNA-Off and UV sterilization prior to and after all eDNA work.**
4. **Turn on the incubator and set the temperature to 56°C. Set the shaker setting to low speed (150rpm). Allow to come up to temperature before continuing.**
5. **Place the Buffer ATL into the shaker in order to dissolve into solution any precipitate that has formed. This is only necessary if solutes have formed**
6. **Create an extraction blank**
   - **A single 47mm glass fiber/CN/supor filter, rolled and placed into a 1.5 ul microcentrifuge tube, as environmental samples.**
7. **Add 370 ul of Buffer ATL and 30 ul of Proteinase K to each 1.5 ul microcentrifuge tube containing a filtered sample. Vortex immediately and vigorously for 15 seconds. Place samples into a clean tube tray and into shaker incubator.**
8. **Incubate for 1 hour (timed)**
9. **Clean workstation with 50%bleach, DNA-off and UV sterilization before continuing.**
10. **Pre-label all tubes during incubation:**
    - **1 spin column + collection tube per filter**
    - **1 x 1.5 ul tube for lysis collection**
    - **1 lyse & spin basket per filter**
    - **1 x 1.5 ul tube for final elution**
    - **Set aside 3 X 2ul collection tubes per filter (no need to label)**
11. **Prepare work station with 1 sterile forceps per filter. Have hazardous waste bag/vessel prepared for containing used forceps.**
12. **Using sterile forceps, press the lysed filter down to expel some of the lysis solution. Using a 1000ul pipette, draw off as much of the lysis as possible, depositing it into a sterile labeled 1.5 ul tube. Seal the tube, this lysis will be used.**
13. **Using the same forceps, remove the filter and place into the basket (spin column) of the Lyse & Spin basket. If there is any residual lysis, pipette and expel into the 1.5ul tube containing the rest of the lysis.**
14. **Repeat steps 12+13 for all filters.**
15. **Spin all Lyse & Spin baskets at maximum speed for 2 minutes. When complete the filters should appear white and dry.**
    - **If the filters have not given up all the lysis, they may be re-spun. If the second spin isn’t sufficient, a new spin basket may be used. All lysis must be retained.**
16. **Transfer the lysis into the collection tube so all lysis for each filter is within a single tube. Discard filter and basket.**
17. **Add 200 ul of AL buffer and 200 ul of 100% ethanol to each tube. Vortex immediately for 15 seconds.**
18. **Transfer 650 ul of the liquid mix to a labeled DNeasy Blood and Tissue spin column. Repeat for all samples.**
19. **Spin tubes for at 6000xg (8000 rpm) for 1 minute.**
20. **Discard flow through. Repeat steps 18+19 until all liquid is run through spin column.**
21. **Add 500ul of AW1 buffer. Spin at 6000xg (8000 rpm) for 1 minute. Discard flow through and place spin column into a fresh collection tube.**
22. **Add 500ul of AW2 buffer. Spin at maximum speed for 2 minutes. Discard flow through and place spin column into a fresh 2ul collection tube.**
23. **Spin at maximum speed for 1 minute. Discard flow through carefully, ensure spin column does not contact any flow through at this point. Change to a fresh sterile collection tube.**
24. **Add 100 ul of AE Buffer. Incubate without spinning at 56°C for 5 minutes.**
25. **Spin at 6000xg (8000 rpm) for 1 minute.**
26. **Discard spin column. Transfer flow through from 2 ul collection tube to labeled, sterile 1.5 ul microcentrifuge tubes.**
27. **Store DNA extract at -20°C for short term use or -80°C for archival or long term storage.**
28. **Clean entire work station with 50% bleach solution, DNA-off and UV sterilization upon completion. Clean pipettes with DNA-off. Soak tube trays and forceps in 50% bleach solution for a minimum of 10 minutes, then rinse thoroughly with RO/DI water. Forceps should be packed for autoclaving and autoclaved at convenience. Items should finally be UV sterilized in the cabinet sterilizer.**

**E. QUALITY CONTROL**

1. Extraction negative should not amplify in final PCR results
2. **VARIATION:** Some variation in protocol may be introduced for different filter types. EXAMPLE: Cellulose nitrate and supor filters may not require lyse and spin baskets. Some lysis will be lost, but minimal, and may be acceptable for CERTAIN projects.

EXAMPLE: Incubation times may be altered for each project, but must be standard within a project.

EXAMPLE: Buffer AL and EtOH volumes may be increased if lysis is of high volume (from filters that were not properly dried during filtration).

1. **NOTES FOR eDNA WORK:**
   1. **Gloves should be changed regularly. At any point if user has touched the inside of a cap or a potentially contaminated area, gloves must be changed. Ensure attention to detail in this area, and be especially cautious of not contaminating reagents and equipment. Filter transfer is the most critical step in terms of cross-contamination between samples and an unclean workstation.**
   2. **Reagents must be treated carefully. Do not re-use tips. Always change tips.**
   3. **Developing a work plan before starting is vital. Set up workspace in a way that is easy and comfortable, but most importantly will decrease the likelihood of contamination. Be prepared before you start.**
   4. **Ensure that any bleach on the bench top is dry before resuming work.**
